# Opportunity or Orientation? Who Uses Urban Parks and Why

**DOI:** 10.1371/journal.pone.0087422

**Published:** 2014-01-29

**Authors:** Brenda B. Lin, Richard A. Fuller, Robert Bush, Kevin J. Gaston, Danielle F. Shanahan

**Affiliations:** 1 Climate Adaptation Flagship/Marine and Atmospheric Research, Commonwealth Scientific and Industrial Research Organisation, Melbourne, Victoria, Australia; 2 School of Biological Sciences, University of Queensland, Brisbane, Queensland, Australia; 3 School of Population Health, University of Queensland, Brisbane, Queensland, Australia; 4 Environment & Sustainability Institute, University of Exeter, Penryn, Cornwall, United Kingdom; 5 School of Biological Sciences, University of Queensland, Brisbane, Queensland, Australia; University of California, Berkeley, United States of America

## Abstract

There is growing recognition that interactions with nature provide many desirable human well-being outcomes, yet increasing urbanization is degrading the quality and quantity of nature experiences. Thus, it has become increasingly important to understand how and why urban dwellers interact with nature. Studies of urban green space use have largely focused on the availability and ease of access to green space, suggesting that greater *opportunities* to experience such space will lead to increased use. However, a growing literature emphasizes the potential for an individual's nature *orientation* to affect their interaction with green space. Here we measure the importance of both opportunity and orientation factors in explaining urban park use. An urban lifestyle survey was deployed across Brisbane, Australia in November 2012 to assess patterns of green space use. Participants (n = 1479) were asked to provide information on demographics, private yard use, park visitations in the past week, and their orientation toward nature. About 60% of those surveyed had visited a park in the past week, and while this park user population had significantly greater nearby park coverage (within a 250 m radius; p = 0.006), a much stronger determinant of visitation was their higher nature orientation (p<0.00001), suggesting that while both opportunity and orientation are important drivers for park visitation, nature orientation is the primary effect. Park users also spent significantly more time in their yards than non-park users (p<0.00001), suggesting that yard use does not necessarily compensate for lower park use. Park users with stronger nature orientation (i) spent more time in their yard, (ii) traveled further to green spaces, and (iii) made longer visits than park visitors with weaker nature orientation. Overall, our results suggest that measures to increase people's connection to nature could be more important than measures to increase urban green space availability if we want to encourage park visitation.

## Introduction

A major change in the human-ecological landscape is the dramatic shift to urbanization, with more people concentrated in cities [1]. Urban dwellers now exceed 50% of the global population, and urban areas are predicted to absorb the majority of the continued population growth over the next four decades [2]. Cities are inevitably relatively nature-poor due to the great range of competing land-uses. Because of this and the fact that urban residents are leading increasingly busy lives, there is concern that people are becoming disconnected from nature, leading to a large-scale extinction of experience with the natural world [3], [4]. This extinction of experience could have important consequences for the well-being of urban populations [5]. Exposure to and interaction with nature have been shown to have a role in physical health, cognitive function, social cohesion, and mental health, with long-lasting psychological benefits [6], [7], [8], [9]. Furthermore, urban green spaces provide arenas for recreation, community activities, and physical activities, with the last being a significant protective factor from cardiovascular disease, diabetes, and obesity [10], [11], [12].

With a growing recognition that interacting with nature is important for many human and environmental health outcomes [5], it has become increasingly important to understand what drives the extent to which urban dwellers interact with the green space around them [13], [14]. As a possible catalyst for physical activity, the prevailing view is that the provision of clean, safe green spaces is particularly important [15], [16], [17]. Furthermore, the provision of good quality green space is considered a possible mechanism for tackling health inequalities [18], [19]. This has led to governments and city councils setting minimum area targets for the provision of parks (i.e. public green space) and to reduce any impediments associated with the use of parks [20], [21]. Such targets and actions rely on the concept that providing the *opportunity* for people to use parks close to where they live and work will itself result in their use.

However, a focus on proximity and access to parks might poorly capture other social dimensions that drive park usage, and so improving access alone may be insufficient to result in greater park use and the associated well-being benefits [22], [23]. Individual factors, such as motivation to engage in outdoor activities or orientation towards nature, may be equally very strong drivers of park visitation behavior [24], [25]. In this case, it is an individual's *orientation*, rather than *opportunity* alone, that may determine the motivation to spend time in urban green space. If orientation is an important motivator for park visitation, the benefits of the interaction would mainly be gained by people with a high orientation towards nature and nature experiences, and those with a low orientation may be less frequent visitors to parks.

Understanding how both *opportunity* and *orientation* influence people's visitation of parks is essential if we are to design cities where urban dwellers can receive the multitude of well-being benefits provided by these spaces. Here, we measure the drivers of human interaction with parks in Brisbane, Queensland, a rapidly growing city in sub-tropical Australia. A survey of 1479 people was conducted in 2012 to understand better the profile of urban-dwellers who use parks, how they differ to those who do not visit parks, and to assess the relative importance of opportunity and orientation in driving people's green space visitation behavior. We expected to find that park visitation is driven principally by opportunity, with nearby parks visited more often due to convenience. However, individuals who have a high orientation toward nature may be more frequent visitors to parks. We also investigated whether park visitation is attenuated by the time that home owners spend in their own yards since their close proximity makes them easy to access.

## Methods

### Site description

**Figure 1:**
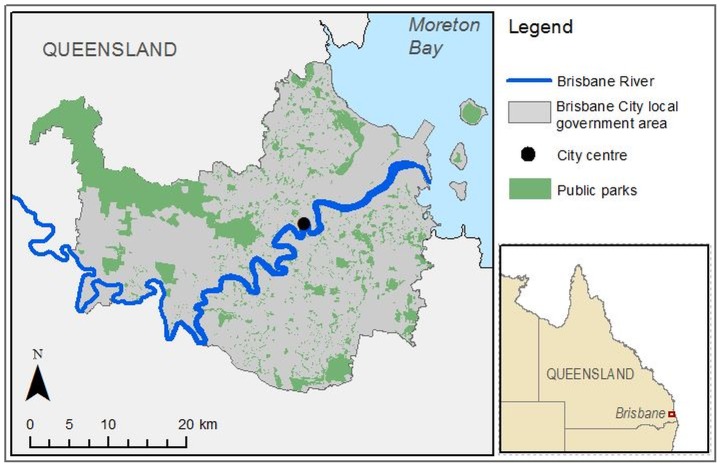
Map of Brisbane with survey area (in gray) and parks (in green).

Brisbane is a subtropical city located in Queensland, Australia (Figure 1). The city government area covers 1380 km^2^ and in 2011 had an estimated population of 1,090,000 residents. The region has significant projected population growth, with 156,000 additional dwellings forecast to be required within the greater Brisbane area by 2031 (up from a total of 397,000 dwellings in 2006 [26]). There is currently significant sprawling development on the outskirts of the city, but the majority of the population is concentrated in a strip through the center. Brisbane is a city with considerable amounts of green space per capita, and public green spaces and parks are distributed rather evenly across the city.

### Survey data collection

#### Ethics clearance

This research was conducted in accordance with Institutional Human Research Ethics Approval (Behavioural & Social Sciences Ethical Review Committee, University of Queensland), project number 2012000869. Because the survey was administered electronically, participants were asked to provide written consent by checking a box stating their agreement to participate in the survey. On the written consent form, participants were told that their data would remain anonymous and would be protected and stored in a secured format. There is an electronic log of the consent procedure to document the process. Participants were compensated a nominal fee for their participation in the survey. This procedure was also approved by the Behavioural & Social Sciences Ethical Review Committee, University of Queensland.

#### Participants and procedure

An urban lifestyle questionnaire was delivered to participants across Brisbane in November 2012 (n = 1479) by a market research company to a stratified sample of people enrolled in a survey database. The market research company had access to a wide range of potential participants representing the general population of Brisbane. We sampled with strata to ensure that the respondents reflected a range of demographic groups, as well as a wide spread across the socio-economic gradient within Brisbane, namely that: (i) all participants were between 18 and 70 years of age, (ii) an equal number of participants were above and below 40 years of age, (iii) an equal number of participants were male and female, (iv) income quartiles of the participant group reflected those of the total Brisbane population (based on the 2011 Australian Census data), and (v) participants were spread evenly across four spatial zones that reflect the four quartiles of available tree cover in the city.

We conducted the survey in early summer (November), matching the seasonal timing of health surveys in Australia where outdoor exercise and park usage are important variables [27]. This represents a time when people may be more likely to use outdoor spaces before the higher temperatures of late summer become a deterrent. Participants were asked to provide information on age, gender, and educational qualifications. Although the participants were not asked to identify their race, participants were asked the primary language spoken at home. A primary language other than English was spoken by 13% of respondents, with 47 languages being spoken across the sample group as a whole. Preliminary analysis to determine whether primary language had a large effect on park usage did not reveal any statistically significant differences, and thus primary language was excluded from further analysis.

Each participant was also asked to provide their address or the location of the street corner closest to their home if they did not wish to reveal their precise street address. Participants were asked to report on whether they had visited a public park in the past week, and to provide either the name, location, or some identifiable land mark that could assist us in locating the park on a map. Participants indicated the amount of time they had spent in each park they had visited. To better understand the possible moderating effect of residential yards on park visitation, participants were also asked to report the time they spent in their own yards over the past week.

### Analysis – opportunity versus orientation

#### Orientation measurement

Survey participants were asked to complete the Nature Relatedness Scale (referred to as ‘NR’ here) [25]. This score was used to indicate the strength of each participant's orientation towards nature. This scale requires participants to complete a series of questions that assess the affective, cognitive, and experiential relationship individuals have with the natural world [25]. Participants rate 21 statements using a five-point Likert scale ranging from one (disagree strongly) to five (agree strongly). Responses to each of the 21 questions were scored and then the average was calculated according to the system outlined by [25]. A higher average score indicates a stronger connection with nature. The scale has been demonstrated to differentiate between known groups of nature enthusiasts and those not active in nature activities, as well as those who do and do not self-identify as environmentalists. It also correlates with environmental attitudes and self-reported behavior and appears to be relatively stable over time and across situations [25].

#### Opportunity measurement

Each participant's opportunity to visit parks was measured as the area of park available within a 250 m, 500 km, and 1 km radius around their home. To measure this, we developed a GIS layer that identified all publicly accessible parks in Brisbane using information from the Brisbane City Council and the Queensland Government.

#### Park vs. non-park user comparison

We split the respondents into two groups: people who had used parks in the previous week and people who had not.

To examine the relative importance of opportunity and orientation, we examined differences in the availability of park space around the home (as measured by park area within 250 m, 500 km, and 1 km radii around the home), the orientation of people based on their NR score, and time spent in yards in the past week as a potential moderating factor for park visitation. Differences between the two groups were assessed using a t-test between means.

A general linear model was also run across the entire set of respondents (response variable was a binary indication of park use or non-park use) to understand the relative importance of opportunity and orientation variables on park visitation. The predictor variables for opportunity were % park cover (at 250 m, 500 m, and 1 km radii around the home, each tested in a separate model), NR score (as a measure of orientation), time spent in yard (as a possible moderating factor), and demographic factors of education and age.

#### Park visitation behaviour

Next, we narrowed our analysis to concentrate on the park user subset of respondents. Although park users are the subset that are gaining the benefits of nature interaction, there is still a large spectrum of difference across this group, with some individuals spending significantly more time and travelling to many more parks than others, and thus, potentially receiving different levels of well-being benefits.

We calculated the total time each respondent spent in parks in the past week as an indication of the level of use. We also calculated the total distance that each individual traveled to visit parks based on their park visitation data in the past week. The driving distance between the home address of each respondent and each park visited was estimated using Google Maps. This allowed us to investigate willingness to travel to parks and how this varied with differing levels of park availability near the home, the amount of time spent in yards, and the nature relatedness score.

Two response variables were used for this analysis: ‘total distance traveled to parks’ and ‘time spent in parks’. Linear regression models were run using the predictor variables % park cover (run separately for each of the 250 m, 500 m, and 1 km radii park area measurements around the home), NR score, and time spent in yard.

All spatial analyses were carried out in ArcGIS (v10.0). All statistical analyses were carried out in R v2.13.0 [28]. Variables were transformed where appropriate to more closely conform with assumptions of normality.

## Results

### Park vs. non-park user comparison

**Figure 2:**
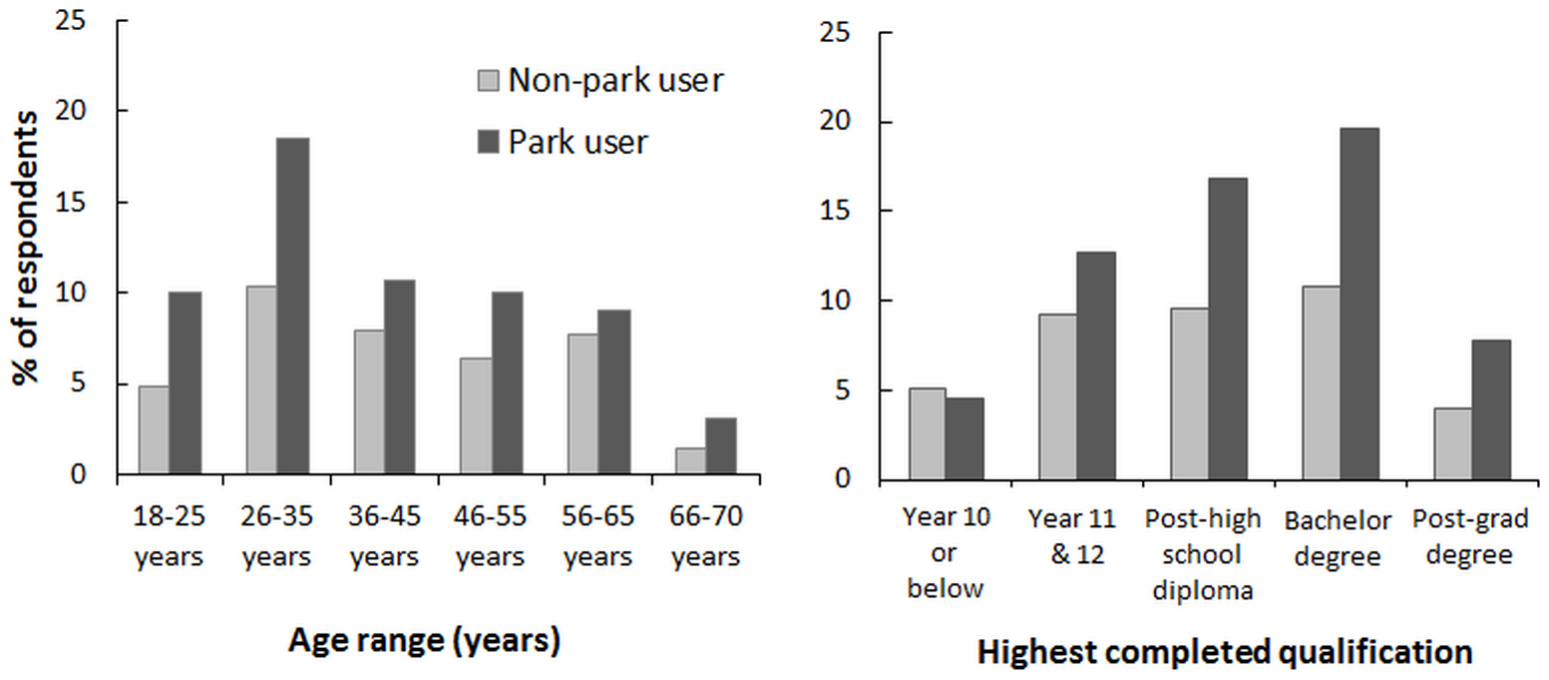
Differences in age and educational qualifications between non-park and park users: genders were approximately evenly represented in both groups, but park users (red) were slightly younger and older than non-park users (blue) and had completed more educational qualifications.

Sixty-two percent of survey respondents had visited a park in the past week (n = 914) and 38% had not (n = 565). Overall the gender split was almost equally 50%:50%, male to female in both populations, although it was slightly more skewed to males in the park users group (54%:46%). Park users were also slightly younger and had a higher level of formal education than non-park users (Figure 2).

**Figure 3:**
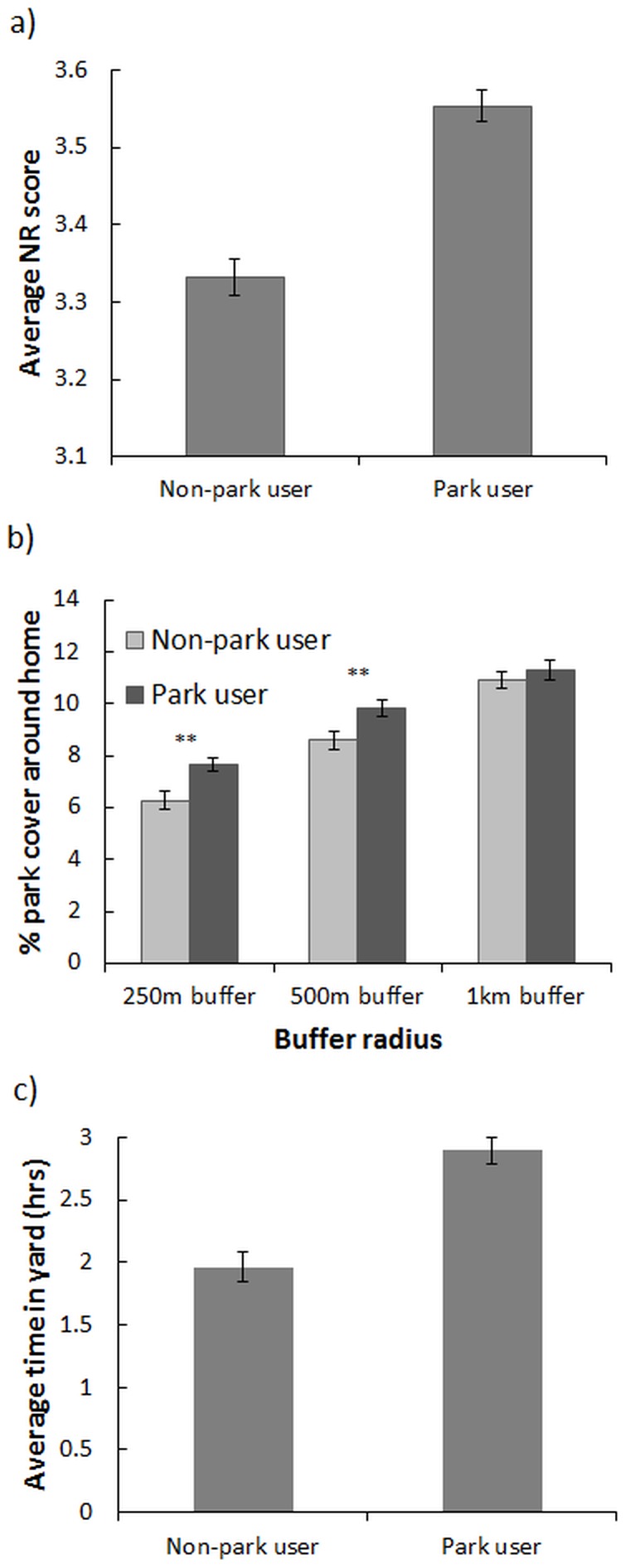
Comparison between non-park and park users of a) participants' nature relatedness (NR) score, b) coverage by parks at a 250 m, 500 m, and 1 km radius around the home, and c) average time spent in private yard in one week. Park users showed significantly higher levels of nature relatedness (p<0.00001), had greater park coverage close to their homes (250 m and 500 m), and spent more time in their yards than non-park users (p = 0<00001; significance codes: 0.05*, 0.01**, 0.001***).

Park users had more parks available around their homes at radial distances of 250 m (t = −2.86, p = 0.004) and 500 m (t = −3.17, p = 0.001; Figure 3), but not at 1 km (t = −1.57, p = NS). Park users also spent more time in their yards than non-park users (t = −6.48, p<0.00001) (Figure 3), indicating that yard use was not necessarily compensating for lower park use in the non-user group. Mean nature relatedness was much higher among park users than non-park users (t = −7.22, p<0.00001; Figure 3).

The generalized linear models (GLM) examined the extent to which opportunity and orientation variables, as well as the moderating factors of time spent in yard, age, and education, were correlated with use and non-use of parks at the three different spatial scales (see Table 1 for the results). At the 250 m and 500 m scale, park cover (opportunity), NR (orientation), and time spent in yard (moderating) were significant, however with different levels of effect. At both scales, nature relatedness had the highest β coefficient (β = 0.570 and 0.569, respectively) signifying the greatest level of effect. Time spent in yard had slightly lower β coefficients (β = 0.346 and 0.418, respectively), and park availability had the lowest (β = 0.133 and 0.182, respectively), signifying a lower level of effect of the opportunity variable. In the third model, at the 1 km scale, park cover (opportunity) was not significant, but NR score (β = 0.576) and time spent in yard (β = 0.419) were still important explanatory variables (Table 1). This signifies that the effect of park cover becomes less important than NR at larger scales. This supports our earlier analysis that park availability beyond 500 m does not explain differences in park use, perhaps because park availability becomes rather even at this coarse scale. This analysis also suggests that time spent in yards is higher for park users (rather than the earlier hypothesis that it would be higher for non-park users), and NR scores are highly indicative of green space use. In all analyses, age and qualifications were relatively weak predictors of park and non-park use.

**Table 1:** Results from generalized linear model analyses examining the relationship at three different spatial scales between opportunity (nearby park coverage), orientation (NR score), time spent in yard (as a moderating factor), and age and qualifications on the binary response variable Park/Non-Park Use.

Predictor variables β coefficient (z-value)							
Buffer size	% Park cover	NR score	Time spent in yard	Age	Qualifica-tions	?^2^	AIC
250 m	0.133 (2.85) **	0.570 (5.83) ***	0.346 (5.677) ***	−0.017 (−4.05) ***	0.155 (3.165) **	113.08***	1866.1
500 m	0.182 (3.07) **	0.569 (5.83) ***	0.418 (5.61) ***	−0.016 (−4.04) ***	0.150 (3.07) **	133.08***	1866.1
1 km	0.182 (2.007)	0.576 (5.92) ***	0.419 (5.64) ***	−0.017 (−4.10) ***	0.153 (3.13) **	107.63***	1871.6

Each line represents a different model, with each % Park cover buffer (250 m, 500 m, 1 km) analyzed in a separate model analysis.

The χ^2^ value indicates the difference in deviance between the model and a null model. Akaike's Information Criterion (AIC) is provided to compare between the models with different spatial scales. (Significance codes: 0.01**, 0.001***).

### Park visitation behavior

For the park users, only the NR score was a significant predictor of total distance traveled to parks when tested using linear regression (park availability at 250 m, 500 m and 1 km radial distances and time spent in yard were not significant; Table 2). These results show that orientation is a stronger predictor of the total distance that park users travel to parks rather than the geographic proximity of parks.

**Table 2:** Results from linear regression analysis examining the relationship between Total Distance Traveled to Parks and Time Spent in Parks (response variables) and the predictor variables for opportunity (% Park cover), orientation (NR score), and time spent in the yard as a moderating factor.

Predictor variables β coefficient (t-value)								
Buffer size	% Park cover	NR score	Time spent in yard		Age	Qualifica-tions	R^2^	AIC
**Total Distance Traveled to Parks**								
250 m	−0.040 (−0.96)	0.226 (2.69) **	−0.005 (−0.10)	−0.007 (−2.06) *		0.075 (1.68)	0.019**	2727.2
500 m	−0.093 (−1.69)	0.230 (2.74) **	−0.008 (−0.16)	−0.007 (−2.03) *		0.075 (1.69)	0.022**	2725.3
1 km	0.015 (0.17)	0.224 (2.66) **	−0.004 (−0.09)	−0.008 (−2.16) *		0.077 (1.73)	0.018*	2728.1
**Time Spent in Parks**								
250 m	−0.006 (0.38)	0.191 (5.35) ***	0.087 (3.92) ***	−0.001 (−1.22)		0.044 (2.35) *	0.061***	1780.9
500 m	−0.008 (−0.38)	0.191 (5.36) ***	0.087 (3.91) ***	−0.001 (−1.17)		0.044 (2.33) *	0.061*	1781.0
1 km	−0.016 (−0.45)	0.191 (5.36) ***	0.087 (3.91) ***	−0.001 (−1.14)		0.043 (2.30) *	0.061***	1780.9

Each line represents a different model, with each % Park cover buffer analyzed in a separate model analysis. Akaike's Information Criterion (AIC) is provided to compare between the models with different spatial scales. (Significance codes: 0.05*, 0.01**, 0.001***).

**Figure 4:**
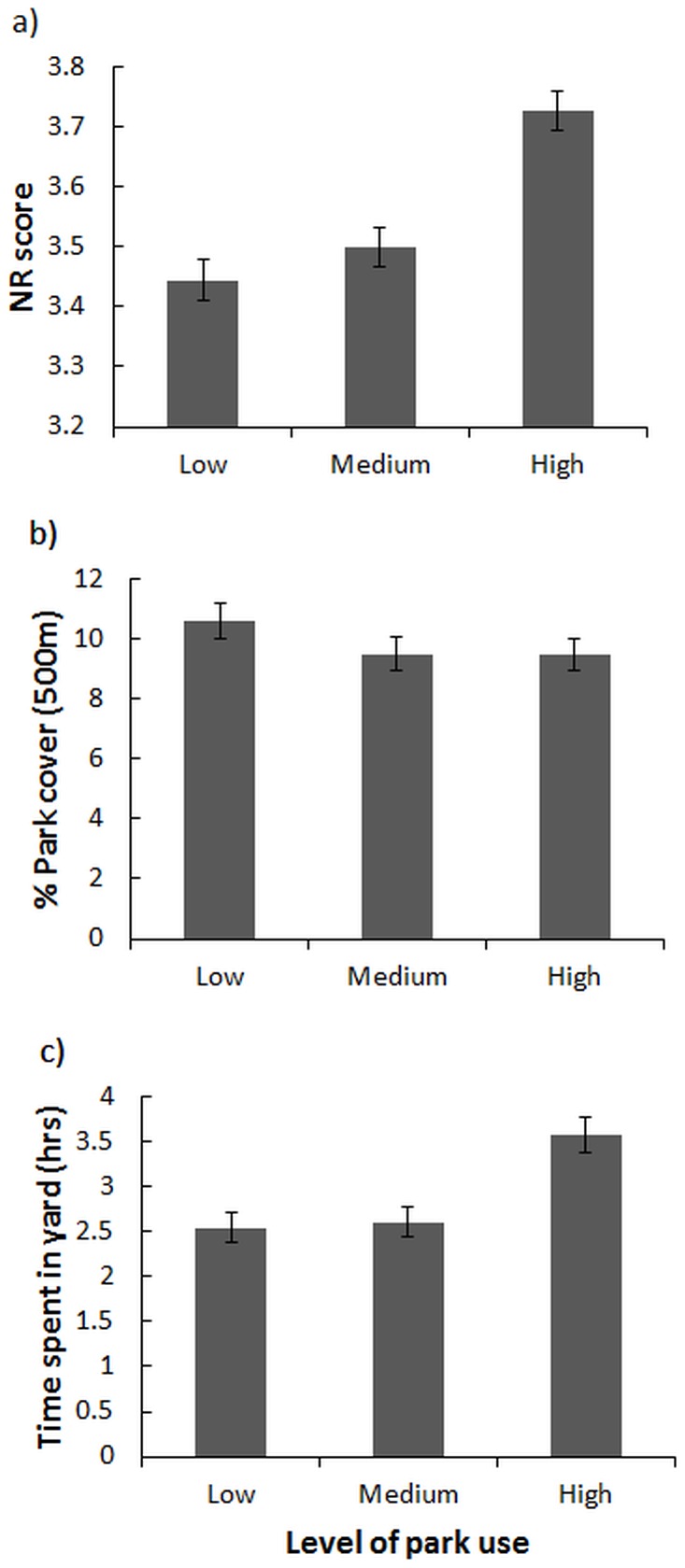
Comparisons of low, medium, and high park users (based on time spent in parks) according to a) participants' nature relatedness (NR) score, b) coverage by parks at a 250 m, 500 m, and 1 km radius around the home, and c) average time spent in private yard in one week.

At all three distances (250 m, 500 m, 1 km), park availability was not an important predictor of time spent in parks, although NR score and time spent in yard were significant, with NR score achieving the highest β coefficient (Table 2). The differences among groups (low, medium, high levels of park use) are illustrated in Figure 4. We conclude that nature orientation is a more important driver of time spent in parks than the availability of green space, and that people who are oriented towards nature will spend more time overall both in parks and in their yard.

## Discussion

With human populations becoming more urbanized, a key emerging global challenge is to provide for both environmental and human well-being needs under severe land constraints [29], [30]. Thus, it will become ever more important to understand how to motivate urban dwellers to spend time in parks so they can gain the range of well-being benefits from green space exposure [3].

Our results reveal that park users, as a group, generally exhibit higher nature relatedness and not only spend more time in parks, but also in their own residential yards (Figure 3). The results also reveal that urban dwellers are motivated to use park space primarily by their *orientation* toward nature, and less so by the *opportunity* to access nearby parks and green space (Table 1 and 2). Thus, the individuals who are visiting parks are significantly skewed toward those who already have a high affinity for and connection to nature. Park users also tend to live in areas that have a higher percentage of parks around their homes (at least within a 500 m radius; Figure 3). Although this may be related to their higher levels of nature relatedness, we are unable to distinguish the direction of cause and effect, i.e. whether people with high NR move to areas with more parks or if the level of nearby green space actually influences an individual's NR.

We initially hypothesized that non-park users might compensate for their lack of park visitation by spending more time in their own yards. However, the opposite was in fact the case, with park users tending to spend more time in their private yards than non-park users. This is an important result because it suggests that planners cannot assume yards and public green spaces are substitutable. Nature orientation was also significantly correlated with total distance traveled to parks and time spent in parks, indicating that people with strong nature orientation traveled further and more frequently to spend time in green space, while park cover was not significant in these analyses. Overall, we have identified a group of individuals that interact with nature to a large degree in both private and public green space most likely because of their high orientation to nature, and not simply because green spaces are available.

Our results suggest that the motivation to visit parks and interact with nature is driven more by nature orientation than opportunity. This discovery is important for urban green space planners as it suggests that a significant group of people might not use local green spaces even if such spaces are available close to their homes (parks or yards). Indeed, non-park users comprised nearly 40% of our survey population. An immediate challenge is to understand how and why people have such different levels of nature orientation, especially because this may influence the extent to which individuals can gain the health benefits of spending time in nature [14], [31]. The non-park users had relatively weak nature orientation, meaning that not only are they less connected to nature, but they may also find nature less valuable or relevant to their lives. Pyle [32] suggests that a decrease in the level of interaction with nature may lead to a decrease in the value and relevance of nature for people. Thus, there is concern that biodiversity conservation may become less relevant to people as much of the world's population has more limited access or desire to experience natural systems in urban landscapes [33]. Increasing urbanization has also led to greater homogenization of urban ecosystems, perhaps leading to a ‘shifting baseline syndrome’ where people continually ratchet down their expectations of the quality and ecological function of natural areas because they are no longer exposed to high quality natural areas [34], [35]. Additionally, people in urban areas are more likely to encounter urban uniformity in the nature they experience, with reduced exposure to non-urban flora and fauna [3]. Such limitations to experiencing nature may lead to lower levels of nature orientation in urban communities.

Rosenzweig [36] has proposed that conservation science may have to bring nature to people rather than have people come to nature, especially in urbanized environments, because people are losing contact with nature. It has been suggested that instead of restoring and maintaining natural spaces to be more representative of previously existing habitats, we may need to modify places that are already dedicated to human activities to become more natural [37]. In urban systems, habitat can be managed for species such that people can maintain high quality interactions with nature on an everyday basis [38]. The greater integration of nature into the built environment not only has the potential to foster biodiversity conservation, but also to increase human well-being in urban populations and make the natural world more meaningful in people's lives [3]. However, there must be attention paid to both biodiversity and human sensitivities, as there are cultural viewpoints associated with different landscapes with iconic trees, paths, and other landscape features important to particular locations [39].

On the other hand, feeling emotionally connected with nature predicts environmental concern [40], [41], and it can be argued that encouraging people to be more connected to nature may be important in order to increase human well-being in cities. Indeed, many conservation biologists and ecologists have recognized that science alone is not sufficient to bring about conservation, rather public education and policy will become increasingly important to protect biodiversity [37]. This may require nature awareness activities that allow people to directly interact with nature and perhaps at a young age to develop deeper nature connectedness [42]. Educational theory also suggests that biophilia and nature orientation is encouraged by early experiences with nature [37]. Thus, urban populations will require better science education and increased nature experiences in order to develop nature connectedness, especially by providing firsthand experiences for children to interact in nature spaces and examine biological elements. This may also require children to have unscheduled time in which they can explore the outdoors in safe places [43]. Pyle [32] suggests that areas of undeveloped or unmanaged land within walking or biking distance may allow children to realize their potential for self-teaching. Research also indicates that children who play in wild environments show a greater affinity and appreciation for such places in later life.

If, as our data suggest, nature orientation is a strong driver of nature experiences and the well-being benefits that flow from them, then urban greening policies must go well beyond spatial urban planning and also focus on understanding and enhancing nature orientation. Future research to understand how nature relatedness is developed in individuals will be necessary to show how interventions can be designed to improve experiences of nature for our urban populations.
